# Reviewing Teledentistry Usage in Canada during COVID-19 to Determine Possible Future Opportunities

**DOI:** 10.3390/ijerph19010031

**Published:** 2021-12-21

**Authors:** Sonica Singhal, Shwetabh Mohapatra, Carlos Quiñonez

**Affiliations:** 1 Public Health Ontario, Toronto, ON M5G 1M1, Canada; 2 Department of Dental Public Health, Faculty of Dentistry, University of Ontario, Toronto, ON L1H 7K4, Canada; carlos.quinonez@utoronto.ca; 3 Faculty of Science and the Schulich School of Medicine & Dentistry, Western University, London, ON N6A 3K7, Canada; smohapa4@uwo.ca

**Keywords:** teledentistry, environmental scan, virtual care, access to care, COVID-19, adjunct service

## Abstract

During the COVID-19 pandemic, the limited in-person availability of oral health care providers resulted in an unprecedented utilization of the teledentistry tool. This paper reviews how Canadian organizations supported teledentistry and what can be expected about its usage in the post-pandemic era. An environmental scan across relevant Canadian federal, provincial, and territorial organizations was conducted to review pertinent publicly available documents, including dental regulators’ or associations’ COVID-19 guidance documents, government documents, and media articles. Almost all jurisdictions promoted teledentistry for triaging dental emergencies and screening patients for COVID-19 symptoms but not even half of them have developed guidelines in terms of modalities of usage, handling of personal information, informed consent process, or maintaining standards of practice. During the COVID-19 recovery phase, these advances across Canada will support in developing a comprehensive guidance for teledentistry and possibly specific codes for its utilization. This can create a niche for teledentistry as an adjunct to the main stream dental care delivery where some visits can always be accommodated virtually, reducing disparities in oral healthcare between rural and urban communities. Ultimately, this can potentially make oral health care delivery more effective, efficient, and environmentally friendly in Canada.

## 1. Introduction

According to the Merriam Webster dictionary, “tele” means distant; anything performed from a distance or remotely is tele, such as television, telephone, or telegraph [1]. In the health care industry, telemedicine or telehealth are commonly used terminologies. A more precise term in the field of dental care is “teledentistry”, essentially a subset of telemedicine, a combination of electronic information, telecommunication technologies, and dentistry [2]. It includes the exchange of clinical information and images remotely for dental consultation, treatment planning, and education [2]. This virtual mode of delivering dental care was first used by the National Aeronautics and Space Administration [NASA] in 1970s and then by the US military [3]. The term teledentistry, however, was first used in 1997 by Cook who defined it as “the practice of using video conferencing technologies to diagnose and provide advice about treatment over a distance” [4]. As such, the history of virtually transferring information goes back thousands of years. The ancient Greeks and Native Americans used smoke signals or heliographs to warn nearby tribes of bubonic plague ravaging in areas or sometimes about war zones [5].

Before the COVID-19 pandemic happened, teledentistry was being used more in public programs than in private care. A number of strengths and weaknesses were considered around its usage. One of the biggest strengths identified was addressing the unavailability of a dental professional in close vicinity, and reducing travelling long distances to receive dental care, especially in rural and remote regions [3,4]. It was also observed that it reduces the cost for both patient and the provider; the patient does not need to invest time and money in travel, or have to pay for child care or take leave from their job for an appointment, and, for the provider, it is time efficient as no chair preparation time between patients is required, resulting in accommodating more patients in a day, and would probably result in fewer no shows [6,7,8]. Video conferencing or using recorded videos virtually are also considered effective in educating people about practicing oral hygiene. In addition, teledentistry is identified as a resource for dental professionals in treating their patients, for example, general dentists can share the records with dental specialists to have their opinion on cases and can, accordingly, decide to treat themselves or to refer [9].

As there are a number of strengths, there are some weaknesses too. One of the distinct limitations with the virtual mode of delivering dental care is that one can perform limited activities virtually—including screening, consulting, history taking, and triaging; most patients need a procedural activity along with examination, which can be performed in clinic only. Moreover, based on what equipment or technology is used for teledentistry, it could require the purchase of additional equipment and training of staff, which can be challenging as not everyone is comfortable using software and sophisticated tools. As for patients, it is not the same experience as discussing problems in person with a professional. This can be further inhibiting if the virtual provider changes every time, which can disrupt continuity of care [10].

On 11 March 2020, the World Health Organization declared the global outbreak of COVID-19 a pandemic [11], and within days, most Canadian jurisdictions, to contain the spread of infection, enacted a declaration of emergency to protect the public. For the dental world, all non-essential and elective dental services were also suspended and only emergency treatment was allowed to continue, which is basically life-threatening conditions (March 2020–May 2020). For urgent conditions, where someone might experience severe dental pain from pulpal inflammation or because of extensive caries, abscess or localized bacterial infection, or tooth fracture resulting in pain, there was limited availability of dental care. As such, in dentistry we come across more urgent conditions than emergent, and though non-life threatening, the individual who is facing that situation is in dire need of care. The early months of the COVID-19 situation made people with urgent dental conditions, irrespective of their social or economic strata or where they lived, vulnerable and disadvantaged because of the limited availability of timely dental care.

During that time, teledentistry emerged as a useful tool; a surge in the widespread usage of this modality across Canadian jurisdictions to address patients’ dental needs was observed. Dentists with the feeling of responsibility towards their existing patients and bounded by their legal and professional obligations started using various virtual modes to have consultations to assess and triage patients, calling in a prescription for appropriate medications, or making referrals to specialists when needed. That said, they faced challenges in understanding the privacy risk of using a virtual care tool, obtaining informed consent remotely, or ensuring record keeping met regulatory requirements because teledentistry was not a commonly used tool, and there were no specific guidelines as to how to use teledentistry in a private dental practice. Dental professionals were not sure what specific codes to use from fee guides for providing care virtually, and how much to charge for providing such care. Additionally, there was no clarity if insurance companies would be covering the cost or not, and if yes, how much.

In response, some Canadian jurisdictions did develop some guidelines around teledentistry, but, moving forward, if we want teledentistry to be utilized to its full potential, we need comprehensive guidance around its usage, which can be utilized all across Canada. The first step would include conducting an environmental scan to review all pertinent literature to understand how the government and other organizations have promoted teledentistry and virtual health care in general since the COVID-19 pandemic happened. This information would support in assessing the opportunities for making teledentistry a crucial armamentarium for dental professionals in Canada to address oral health problems of their patients in a timely manner.

## 2. Methods

An environmental scan was planned across all Canadian jurisdictions. Such a scanning process supports in assessing the internal strengths and challenges and external opportunities and threats of any program or intervention. Such data are used to guide decision making and strategic planning [12,13]. We conducted this scan during July–August 2021. As inclusion criteria, we included publicly available documents across relevant federal organizations of Canada and all provincial and territorial jurisdictions, for example, dental regulators’ and/or associations’ COVID-19 guidance documents, government documents published about the usage of teledentistry in the context of COVID-19, and media articles. Documents were reviewed to assess if there was any specific information about the ways teledentistry is being used or recommended, for example, patient consultation, screening for dental problems and/or COVID-19 symptoms, triaging patients based on treatment urgency, or emergency care. To understand the barriers for the usage of teledentistry, we reviewed and analyzed whether any exceptions/rules were mentioned in organizations’ documents for using teledentistry. We also contacted various insurance companies covering dental care to understand the coverage for teledentistry. Apart from teledentistry specifically, we also reviewed the literature about virtual health care in general; how it has been promoted across Canadian jurisdictions during COVID-19, so that, moving forward, possible opportunities can be explored to determine the scope and utility of virtual modalities in providing dental care in Canada. In terms of the exclusion criteria, the literature from outside of Canada was not reviewed. Additionally, no anecdotal evidence from dental care professionals in terms of the usage of teledentistry or barriers in its usage were included.

## 3. Results

In total, 34 resources were retrieved, of which 21 were related to teledentistry and 13 about general virtual health care. For teledentistry, eight documents were related to federal organizations and the remaining 13 (including three media articles) provided information on provinces and territories. Teledentistry results are presented first (federal followed by provinces and territories) and then those for general virtual health care.

### 3.1. Teledentistry Considerations among Federal Organizations

#### 3.1.1. Government of Canada

Under the Public Health Agency of Canada, the Office of the Chief Dental Officer of Canada released a high-quality review concerning key issues to support dental care providers in delivering safe oral health care in Canada during the COVID-19 pandemic [14]. The report included information on teledentistry as well as from two systematic reviews considering it as a potential means to reduce disease transmission while managing patient care. The report reinforced that telehealth was an appropriate modality to provide continuous care by reducing physical contact and COVID-19-related morbidity and mortality [15]. Other positive impacts acknowledged were the increased acceptance by patients, caregivers, families, and health care facilities; reduced in-person appointments; remote triaging of the elderly [16]. Additionally, it was recognized that virtual care is particularly useful for the evaluation and follow-up of cases that require constant monitoring and, also, the management of patients who are at risk or with symptoms of COVID-19 [17].

#### 3.1.2. Canadian Dental Association (CDA)

On 27 March 2020, just days after the lockdown due to COVID-19 was imposed all across Canada, the CDA published a newsletter stating that the right fee and code for teledentistry was being discussed that could be used consistently across Canada [18]. According to the fourth issue of Volume 7 of CDA Essentials, the CDA was working to facilitate code 05200 for teledentistry and was assisting provinces to implement the code across Canada [19]. According to the same source, some insurance companies had begun adding 05200 to plans or were mapping it to codes already covered concerning adjudicating claims [18]. As per a CDA newsletter published on 17 April 2020, the CDA provided the information to the Canadian Health and Life Insurance Association to enable the insurance companies providing dental plans to add the designated teledentistry codes to plans [20]. The CDA also implemented this code to be covered under the Non-Insured Health Benefits (NIHB) program for eligible First Nations and Inuit clients across Canada [21].

### 3.2. Teledentistry Considerations among Provincial and Territorial Organizations (from East to West)

#### 3.2.1. British Columbia

The College of Dental Surgeons of British Columbia does not seem to have any guidance document around the usage of teledentistry; however, they have included the Royal College of Dental Surgeons of Ontario’s (RCDSO’s) document in their resources, as a reference document [22]. The British Columbia First Nations Health Authority started to cover teledentistry code 05200 [21].

#### 3.2.2. Alberta

In Alberta, on 8 April 2020, The Alberta Dental Association and College developed the Guidelines for Alberta Dentists for Remote Care during the COVID-19 Pandemic, to facilitate the use of remote technology to reduce patient and dentist in-person contact and prevent unnecessary patient visits to emergency departments and community clinics [23,24]. The use of remote dentistry was authorized only during the COVID-19 pandemic and the state of public health emergency in Alberta and introduced a temporary teledentistry billing code for remote consultation services, 05201 [24]. As per the guidelines, the code could be used for consultations with patients exceeding 7.5 min, utilizing a remote dentistry platform and included verifying patient identity, informed consent, review of medical and clinical history, assessment of the clinical situation, interim diagnosis, remote management, appropriate documentation, and subsequent follow-up calls.

#### 3.2.3. Saskatchewan

Nothing in particular to teledentistry usage could be retrieved from the College of Dental Surgeons of Saskatchewan’s website.

#### 3.2.4. Manitoba

In the summer 2020 edition of the Manitoba Dental Association (MDA) bulletin, the inclusion of codes for teledentistry by the CDA was mentioned and teledentistry was used to triage patients from all over Manitoba [25]. The MDA bulletin of spring 2021 mentioned the use of teledentistry to provide a distance-based evaluation of patients in areas with few dental providers and facing difficulties travelling to a clinic, for instance, those in long-term care homes, workers in remote areas, and those with mobility issues [26]. Moreover, the delay in providing dental care in remote regions due to a shortage of dentists and specialists was acknowledged and it was suggested that this could be partially overcome through the digital transfer of information and guiding the less than ideally trained staff who were present in remote areas to extend some level of care.

#### 3.2.5. Ontario

In April 2020, the RCDSO published guidelines for teledentistry to be considered for remote assessment, triage, and extending appropriate dental care while avoiding close patient contact to diminish the risk of transmission of COVID-19 [27]. The acceptable methods included: live (synchronous) audio–visual interaction between a patient and a provider; store-and-forward (asynchronous) mode, where recorded health information of a patient is transmitted to a practitioner for evaluation; remote patient monitoring, where personal health and medical data collection is forwarded to a provider in a different location for use; lastly, mobile health, where public health and education is supported by mobile connection devices such as cell phones, computers, etc. As per the guidelines, teledentistry was to be used by Ontario dentists to treat Ontario patients only. Ontario dentists who practice teledentistry must continue to meet existing standards of practice and the professional, legal and ethical obligations that apply to in-person care. Additionally, teledentistry was only to be used if the risks did not outweigh the potential benefits, and if it was in the patient’s best interest.

On 7 November 2020, the Ministry of Health of Ontario made changes to the schedule of dental benefits in response to COVID-19 and introduced temporary fee codes for hospital-based dentists to create the provision of consultations, follow-up assessments, and visits to admitted bed patients to insured persons (under Ontario Health Insurance Plan) when provided by telephone or video [28].

#### 3.2.6. Quebec

On 1 April 2020, The Ordre des dentistes du Québec (ODQ), regulatory College of Quebec, published guidance on the use of teledentistry [29]. The various methods for the delivery of dental care included live mode, store-and-forward, and remote patient monitoring by a third party [29]. It was recommended to be used only during the state of health emergency declared by the Québec Government to provide emergency dental care to patients who are residents of Québec and to assist in triaging, assessment of patients’ oral health care requirements, and planning of further treatment. It acknowledged the limitations of teledentistry in conducting a full emergency exam and lay the responsibility of following up on the patient’s condition on the dentist who examined, treated, or referred the patient to another dentist.

It was also recommended that the notes in patients’ records should include the technological means utilized, method of identification, and location of the patient during teledentistry appointments. Additionally, the type of consent, whether verbal or written, and the photographs or videos supporting patient complaints, should be included in the patient notes information. Although it gives the dentist the choice of communicating tool for use, it prohibits the usage of any type of social media for communication with patients.

#### 3.2.7. New Brunswick

During the COVID-19 outbreak, the New Brunswick Dental society limited in-person appointments only for emergency procedures and stated that non-urgent care was to be handled strictly by telephone [30]. That said, no public information regarding using specific teledentistry codes was available. Moreover, no guidance document around its usage could be retrieved.

#### 3.2.8. Nova Scotia

The Provincial Dental Board of Nova Scotia developed a COVID-19 Emergency Action Plan, where it mentioned that patients with COVID-19 symptoms should be directed to call 811 for testing [31]. They were to be managed pharmacologically unless life threatening. Other purposes for which the usage of virtual modalities was advised included reminding patients by phone when booking to remove all jewelry from the neck up and any removable dental prostheses. Additionally, they were used to instruct patients to remain in their car or outside the office upon arrival and contact the treatment facility via telephone to check-in. As such, there was no mention of using specific teledentistry codes or other aspects of maintaining specific standards.

#### 3.2.9. Newfoundland and Labrador

The Newfoundland and Labrador (NFL) Dental Board Standards of Practice for Dentistry amended their guidelines in May 2020 by including teledentistry for triaging patients experiencing dental emergencies and screening them [32]. The information and communication technologies included modalities such as synchronous, asynchronous, remote patient monitoring and mobile health, the same as in Ontario. The dentist must protect the privacy and confidentiality of the patient’s health information by using technology under the Personal Health Information Act 2012. The appointment should be held in a private environment after confirming with the patient that they are in a similar setting with secure technology. Appropriate note in patients’ records should be made that the appointment was provided through teledentistry.

#### 3.2.10. Prince Edward Island

No public information from the Dental Association of Prince Edward Island was available in terms of teledentistry codes or around its usage.

#### 3.2.11. Yukon

Although no information could be retrieved from the Yukon Dental Association’s documents mentioning about the codes or standards around its usage, a media article suggests that the President of the Association promoted teledentistry for triaging dental emergencies during COVID-19 [33].

#### 3.2.12. Northwestern Territories and Nunavut

On 23 April 2020, the Executive Director of the Northwest Territories and Nunavut Dental Association, supported giving emergency care to small communities by teledentistry, triaging calls, and using pharmacology [34]. Again, no information was available in terms of teledentistry codes or around its usage.

### 3.3. Coverage through Insurance Companies

We were able retrieve information from four insurance companies through their representatives. As we do not have their consent to identify them, they have been named anonymously.

Company A: The representative shared that all the three teledentistry codes (05201, 05202 and 05209) are covered; however, the coverage varies from plan to plan.

Company B: The representative was not very clear about the coverage and said different insurance plans have different coverage, and the dentist needs to send a pre-determination form to see if the codes are covered. As such, they did not have a list of codes that can be checked with the patients.

Company C: Teledentistry codes were not covered before COVID-19; however, they are currently reimbursing based on special considerations, for example, only for a dental emergency appointment for someone who has or is at risk of COVID-19 and, therefore, cannot attend an in-person appointment. The representative mentioned the coverage would be stopped once COVID-19 is over.

Company D: The representative stated that virtual consultations are not covered. After asking for specific codes, 05201, 05202 and 05209, the representative confirmed the ineligibility.

### 3.4. Virtual Health Care Delivery

Since COVID-19 happened, there has been some major investments being completed in the Canadian landscape in terms of promoting virtual health care delivery. Federal government, with the investment of CAD 200 M, has signed bilateral agreements with provinces and territories for jurisdictions to receive funding to expand virtual health care services [35,36,37,38,39,40,41,42,43,44,45,46]. Dates on which jurisdictions signed the agreement and the respective amount they received are reported in Table 1. No bilateral agreements or funding plans could be retrieved for Quebec.

The bilateral funding is basically to accelerate efforts on virtual care through a focus on three main areas of work [47]: Firstly, the focus is on immediate action in the light of COVID-19; federal, provincial, and territorial Deputy Ministers agreed to five shared priorities for technologies and infrastructure development. These included: “secure messaging and file transfer; secure video conferencing; remote patient monitoring; patient access to their lab results including COVID-19, and back-end supports to enable integration of new tools into existing digital systems.” The second focus area is an evaluation of the impact of virtual care; there is a shared interest in evaluation to assess the impact of virtual services to improve patient care and outcomes, as well as the efficiency and sustainability of care. The third aspect is the development of a federal provincial territorial policy framework, which identifies barriers and opportunities for longer-term adoption of virtual services within Canadian health systems.

The plan is to lay the groundwork to establish virtual services as an additional channel for access to care that complements traditional face-to-face models of care. The policy framework will also be critical to ensuring that governments are well-positioned to address/avoid unintended consequences arising from the widespread uptake and use of virtual care.

## 4. Discussion

The environmental scan suggests that organizations at different levels did support the usage of teledentistry during COVID-19. The CDA was quick, not only in recognizing the utility of teledentistry tool during COVID-19 but also the associated barriers, and, therefore, they identified the specific codes for its usage and also suggested the associated fees. Half of the provinces developed some guidelines around the usage of teledentistry, whether in terms of the modalities of usage, handling of personal information, informed consent process, or maintaining the standards of dental practice as they would be during in-person delivery of care. Almost all jurisdictions promoted teledentistry for triaging dental emergencies and screening patients for COVID-19 symptoms, but it is uncertain how many dental professionals in jurisdictions are actually using teledentistry codes for consultations, examinations, or referrals. Moreover, it is unclear whether dental professionals are able to bill for virtual consultations, as our phone interviews with insurance companies showed that not all, or we should say only a few, have clarity about the 05200 codes and their reimbursement.

It is important to note that in the United States, specific teledentistry codes existed before COVID-19; the American Dental Association in 2018 introduced two codes: D9995 teledentistry—synchronous; D9996 teledentistry—asynchronous [48]. They are reported in addition to other procedures (e.g., diagnostic) delivered to the patient on the date of service. If we had specific teledentistry codes already in the system, their usage to address timely access to dental care could have been further facilitated. More important to understand is that 05200 series codes are for consultation in general and not specific to teledentistry, and they have been used during COVID-19 for virtual purposes as a quick fix. Once we have passed over COVID-19, it is very much possible that these codes are not used again for teledentistry in private practice. Considering the acknowledged importance of including virtual modality to facilitate access to dental care, it will be a missed opportunity window if organized dentistry does not establish teledentistry specific codes in the near future.

In terms of the federal government, it seems that strengthening virtual health care delivery systems in Canada has become a priority in the wake of COVID-19. However, it is important to acknowledge that telemedicine was already in practice in Canada long before COVID-19 and the Canadian Medical Association, along with the College of Family Physicians of Canada and the Royal College of Physicians and Surgeons of Canada was already working on preparing a virtual care guide for patients [49]; this background work probably facilitated these funding agreements. However, there is no allocated funding specifically for dental care, but, that said, overall investment in virtual health care technology gives an opportunity for teledentistry as well, and more importantly, to strengthen linkages between oral and general health care services. As the plan is to lay the ground to establish virtual services as an additional channel for access to care that complements traditional face-to-face models of care, a similar role for teledentistry can also be sought. Accommodating some visits virtually will reduce patient travel, contributing to a reduction in CO_2_ emissions and making it an environmentally friendly option [50]. Moreover, oral health and general health data can be integrated into electronic data reporting systems, so that patients’ complete health information exists in one place, which will support all health care providers including dental to make fully informed treatment decisions for their patients.

Though we can appreciate there a lot of opportunities for improving the usage of teledentistry, at the same time, we need to be mindful of the potential threats associated with delivering care virtually. The same connectivity that makes virtual healthcare possible also creates threats to patients. For example, for achieving a flawless virtual care experience, internet reliability is key, but, in a way, it is a dependence and if for any reason there is no internet, it immediately would affect this delivery model [51]. Another major threat can be data breaches; with increased usage, a lot more data would be stored and transferred electronically through cloud servers or data would be sitting in systems’ hardware, which increases the chances of a data breach [52]. Many platforms do not meet HIPAA (Health Insurance Portability and Accountability Act) requirements and lack adequate data safeguards, which can create cybersecurity issues [52,53]. Additionally, with more and more usage of virtual platforms, people will lose the in-person touch.

## 5. Conclusions: Moving Forward

As we recover from COVID-19, with further development of technological advancements, teledentistry can support us in:

- Improving access and delivery of oral health care with lower costs;
- Eliminating the disparities in oral healthcare between rural and urban communities;
- Enabling people in remote regions to receive dental specialists’ consultations;
- Becoming a complement to routine consults and dental visits, where some visits can always be accommodated virtually;
- Improving inter-professional communications with integration of dentistry into the larger healthcare delivery system;
- Being environmentally friendly by reducing patient travel.

Although we need more studies to assess the effectiveness, cost-effectiveness, and long-term use of teledentistry, the observed strengths and upcoming opportunities indicate that teledentistry may fill a major gap in oral health care delivery.

## Figures and Tables

**Table 1:** Bilateral agreements between the Federal and Provincial/Territorial governments of Canada to receive funding to expand virtual health care services *.

Provincial/Territorial Government (from West to East)	Date of Agreement	Amount Received for Virtual Health Care Services
British Columbia	5 February 2021	CAD 18 M
Alberta	14 April 2021	CAD 16 M
Saskatchewan	1 April 2021	CAD 6.5 M
Manitoba	13 August 2021	CAD 7 M
Ontario	16 April 2021	CAD 46 M
Quebec	N/A	N/A
New Brunswick	6 August 2021	CAD 5.3 M
Nova Scotia	30 March 2021	CAD 5.9 M
Newfound land	6 August 2021	CAD 4.5 M
Prince Edward Island	8 February 2021	CAD 3.5 M
Yukon	29 January 2021	CAD 3.12 M
Northwest Territories	6 May 2021	CAD 3.1 M
Nunavut	26 July 2021	CAD 3.1 M

* This funding is for the general healthcare system and not specific for oral healthcare. As such, healthcare in Canada is primarily publicly funded but dental care is mostly privately funded or covered through employers.

## Data Availability

Not applicable.
